# LanGui tea, an herbal medicine formula, protects against binge alcohol-induced acute liver injury by activating AMPK-NLRP3 signaling

**DOI:** 10.1186/s13020-024-00906-0

**Published:** 2024-03-04

**Authors:** Ming Gu, Yu-jun Chen, Ya-ru Feng, Zhi-peng Tang

**Affiliations:** 1 Institute of Digestive Disease, Longhua Hospital, Shanghai University of Traditional Chinese Medicine, Shanghai, 200032 China; 2 The Third People’s Hospital Affiliated to Nantong University, Nantong, 226006 Jiangsu Province China

**Keywords:** Alcoholic liver disease, Fatty liver, Adenosine monophosphate-(AMP)-activated protein kinase, NLR family pyrin domain containing 3, LanGui tea

## Abstract

**Background:**

LanGui tea, a traditional Chinese medicine formulation comprising of *Gynostemma pentaphyllum* (Thunb.) Makino, *Cinnamomum cassia* (L.) J. Presl, and *Ampelopsis grossedentata* (Hand-Mazz) W.T. Wang, has yet to have its potential contributions to alcoholic liver disease (ALD) fully elucidated. Consequently, the objective of this research is to investigate the protective properties of LanGui tea against binge alcohol-induced ALD and the mechanisms underlying its effects.

**Methods:**

An experimental model of acute alcohol-induced liver disease was performed to assess the protective effects of extract of LanGui tea (ELG) at both 50 and 100 mg.kg^−1^ dosages on male C57BL/6 mice. Various parameters, including hepatic histological changes, inflammation, lipids content, as well as liver enzymes and interleukin 1β (IL-1β) in the serum were measured. The pharmacological mechanisms of ELG, specifically its effects on adenosine monophosphate-(AMP)-activated protein kinase (AMPK) and NLR family pyrin domain containing 3 (NLRP3) signaling, were investigated through Western blotting, qRT–PCR, ELISA, immunohistochemistry, immunofluorescence analyses, and by blocking the AMPK activity.

**Results:**

ELG demonstrated a mitigating effect on fatty liver, inflammation, and hepatic dysfunction within the mouse model. This effect was achieved by activating AMPK signaling and inhibitingNLRP3 signaling in the liver, causing a reduction in IL-1β generation. In vitro studies further confirmed that ELG inhibited cell damage and IL-1β production in ethanol-induced hepatocytes by enhancing AMPK-NLRP3 signaling. Conversely, the pharmacological inhibition of AMPK activity nearly abrogated such alteration.

**Conclusions:**

Thus, LanGui tea emerges as a promising herbal therapy for ALD management involving AMPK-NLRP3 signaling.

**Supplementary Information:**

The online version contains supplementary material available at 10.1186/s13020-024-00906-0.

## Background

Alcoholic liver disease (ALD) is a prevalent global disease that arises from overdrinking [1, 2]. ALD encompasses a range of liver pathologic changes, including steatosis, steatohepatitis, and hepatitis, eventually resulting in liver failure and mortality [1]. The pathogenic factors of ALD involves aberrant lipid metabolism, oxidative stress, endoplasmic reticulum (ER) stress, apoptosis, and cytokine and inflammation [3]. The morbidity and mortality associated with ALD have shown a parallel increase with the rise in alcohol consumption in Western and Asian nations [4]. Nevertheless, little progress has been made in ALD medical care over an extremely long period. Binge drinking, a prevalent manifestation of alcoholism, can lead to acute alcoholic liver injury (AALI) and contribute to the occurrence of ALD-related mortality [5]. However, there has been insufficient emphasis on safeguarding against AALI induced by acute alcohol exposure. Consequently, new strategies are urgently required to mitigate ALD associated with binge drinking.

Adenosine monophosphate-(AMP)-activated protein kinase (AMPK) is crucial for energy regulation [6]. The therapeutic management of metabolic dysfunstions heavily relies on the modulation of AMPK activity [7]. Activation of the AMPK signaling pathway facilitates fatty acid oxidation (FAO) and inhibits de novo lipogenesis, thereby mitigating the fatty liver [7, 8]. Furthermore, AMPK activation attenuates hepatic pro-inflammatory responses, oxidative stress, and liver injury [9, 10], whereas its inactivation exerts the opposite effects [7, 11]. In addition, AMPK activation has been demonstrated to alleviate ALD in mice [12, 13]. Targeting AMPK signaling is a promising option for ALD management.

The NLR family pyrin domain containing 3 (NLRP3) inflammasome can recognizes pathogens and damaged cell signals [14]. When activated, NLRP3 recruits apoptosis-associated speck-like protein containing a CARD (ASC) and caspase-1 (CASP-1), resulting in the cleavage of CASP-1 and subsequent maturation and production of interleukin-1β (IL-1β) [14]. Ultimately, NLRP3 contributes to the inflammatory damage inflicted on the cells [15]. Emerging evidence suggest that the progression of ALD is heavily influenced by the activation of NLRP3 signaling [16]. Conversely, mice lacking NLRP3 demonstrated a beneficial impact on alcohol-induced liver inflammation, as well as a reduction in liver damage and steatosis [17]. Several NLRP3 inflammasome inhibitors have been identified as beneficial in improving ALD [18]. Given the significance of NLRP3 signaling in the pathogenesis of ALD, there has been a growing interest in the pharmacological suppression of NLRP3 signaling.

Interestingly, the activation of AMPK has been found to prevent NLRP3 signaling through various underlying mechanisms, including the regulation of mitochondrial homeostasis, reactive oxygen species (ROS), autophagy, and endoplasmic reticulum (ER) stress [19, 20]. Furthermore, the compound baicalin has been shown to alleviate cerebral ischemia–reperfusion injury by activating AMPK, which in turn inhibits NLRP3 inflammasome activity [21]. Similarly, metformin has been discovered to hinder NLRP3 inflammasome by relying on AMPK, thereby exerting beneficial effects in diabetic cardiomyopathy [22]. Additionally, in mouse models, several activators of AMPK signaling have been demonstrated to attenuate non-alcoholic fatty liver (NAFLD) and ALD by inhibiting NLRP3 signaling and the maturation of IL-1β [23, 24]. Furthermore, there is evidence that the stimulation of AMPK improves liver damage caused by acute pancreatitis, partly by suppressing NLRP3 inflammasome signaling [25]. In contrast, the inhibition of AMPK exacerbates acute liver injury induced by hyperglycemia, as it promotes the activation of NLRP3 inflammasome in liver-resident macrophages [26]. Consequently, the targeting of the AMPK-NLRP3 signaling pathway holds a promise for the management of ALD and AALI.

The LanGui tea formula comprises *Gynostemma pentaphyllum* (Thunb.) Makino (*G. pentaphyllum*), *Cinnamomum cassia* (L.) J. Presl (cinnamon), and *Ampelopsis grossedentata* (Hand-Mazz) W.T. Wang. These three herbs have been recognized as both traditional medicine and functional foods due to their ability to exhibit diverse protective activities against oxidative stress, ER stress, inflammation, liver damage, and metabolic dysfunction [27–29]. Specifically, the active ingredient cinnamic acid found in *C. cassia* (L.) J. Presl favours the recovery from alcohol-induced hepatotoxicity [30]. Moreover, the primary effective ingredient dihydromyricetin present in *A. grossedentata* (Hand-Mazz) W.T. Wang, commonly known as vine tea, has been found to mitigate liver steatosis and injury in alcohol-fed mice by activating AMPK [31]. The vine tea extract rescued mice from non-alcoholic steatohepatitis (NASH) through the activation of AMPK-LXRα signaling [32]. Nevertheless, the potential pharmacological activity of LanGui tea in ALD treatment remains uncertain.

Here, we aimed to explore the beneficial effects of LanGui tea on binge alcohol-induced ALD by regulating AMPK-NLRP3 signaling. We, therefore, assessed the anti-ALD impact and the mechanism of the ethanol extract of LanGui tea (ELG) using in vivo and in vitro ALD models. data showed that ELG effectively mitigated AALI in mice subjected to binge ethanol feeding. Furthermore, ELG was found to activate AMPK signaling, leading to the inhibition of NLRP3 activation in both mouse liver and cultured hepatocytes. These findings suggest that the anti-ALD properties of ELG may be attributed to its modulation of the AMPK-NLRP3 signaling pathway. Overall, our research provides valuable insights into the role of ELG in the protection of ALD.

## Materials and methods

### ELG and chemicals preparation

Dry *G. pentaphyllum* (Thunb.) Makino, *C. cassia* (L.) J. Presl, and *A. grossedentata* (Hand-Mazz) W.T. Wang were purchased from Anhui Huangshan Greenxtract Co., Ltd. (Huangshan, China). ELG was prepared by grinding 450 g of *G. pentaphyllum* (Thunb.) Makino, 100 g of *C. cassia* (L.) J. Presl, and 450 g of *A. grossedentata* (Hand-Mazz) W.T. Wang. Subsequently, the three herbs were combined and subjected to boiling in a 10X volume of 75% ethanol at 100 °C for 2 h for the initial extraction, followed by 1.5 h for the ubsequent extraction. The combined ethanol extract was filtered and concentrated using a rotary evaporator (RE-2000A, Yarong, Shanghai, China) at 50 °C under reduced pressure. After cooling down, the extract was freeze-dried into 100 g of ELG powder using a freeze dryer (LGJ-10N, Yaxingyiqi, Beijing, China). ELG powder was dissolved in dimethyl sulfoxide (DMSO) at a concentration of 100 mg.mL^−1^ for subsequent experiments. Lipopolysaccharides (LPS) and compound C (CC) were purchased from Sigma–Aldrich (St. Louis, MO, United States), with a purity of > 98% for both LPS and CC.

### Ultra-Performance Liquid Chromatography Quadrupole Time of Flight Mass Spectrometer (UPLC-Q-TOF–MS)

ELG powder (250 mg) was dissolved in 25 mL of 75% ethanol. To facilitate dissolution, this solution underwent sonication twice at 300 W and 40 kHz, followed by centrifugation at a speed of 12, 000 rpm for 5 min. The UPLC was performed using UPLC/Q-TOF–MS (Sciex Triple TOF 4600 high resolution mass spectrum coupled with an Agilent 1290 UPLC system; AB Sciex, Darmstadt, Germany; Agilent, Los Angeles, CA, USA). For separation, the UPLC system was utilized with HSS T3 columns from Acquity (2.1 × 100 mm, 1.8 μm; Waters, Milford, CT, USA), and 2 µL of the supernatant was injected. A solvent system comprising 0.1% formic acid-acetonitrile (phase A) and 0.1% formic acid–water (phase B) was used for gradient elution with a flow rate of 0.3 mL/min. The elution program was as follows: 10% A-20% A 0–5 min, 20% A 5–9 min, 20% A-30% A 9–16 min, 30% A-45% A 16–26 min, 45% A-95% A 26–32 min, 95% A 32–36 min, 95% A-10% A 36–37 min, and 10% A 37–40 min. The specific gradient elution program of the mobile phase was illustrated in Additional file 1: Table S1.

### Treatment and culture of cells in vitro

The AML-12 liver cell line derived from mice was obtained from the Shanghai Institute of Biochemistry and Cell Biology, Chinese Academy of Sciences (Shanghai, China). The cells were grown in DMEM-F12 medium (Biological Industry) containing 10% fetal bovine serum (FBS), penicillin, streptomycin, 0.1 mM dexamethasone, and a combination of insulin-transferrin-selenium (ITS). The cells were incubated in a cell culture incubator (Heracell Series, Thermo Fisher Scientific, Thermo Fisher Scientific Inc., Waltham, MA, USA) at 37 °C with 5% CO_2_.

For in vitro ethanol or inflammation modeling and subsequent cell experiments, the AML-12 cells were cultured in DMEM-F12, and then subjected to ethanol (500 mM) [33] or LPS (100 ng.mL^−1^) [34] with or without the addition of DMSO (0.1%), CC (10 μM), and the ELG at specified concentrations for 24 h.

### Animals treatment

The animal experiment protocols were authorized by the Shanghai University of TCM (approval number: PZSHUTCM211115002). All animal experiments were based on the ARRIVE animal experimentation guidelines [35]. C57BL/6 wild-type (WT) male mice, aged 10 weeks and weighing 20–22 g, were obtained from the SLAC Laboratory in Shanghai, China. The mice were kept in a controlled environment with a temperature of 22–23 °C and a 12-h light/dark cycle, ensuring specific pathogen-free conditions. To generate an acute ethanol-binge model, the mice were randomly assigned to the normal WT control (control, n = 7), AALI model (AALI, n = 7), low-dose ELG (50 mg.kg^−1^ i.g.) treatment (AALI + ELGL, n = 7), and ELG (100 mg.kg^−1^ i.g.) treatment (AALI + ELG, n = 7) groups according to their body weights. ELG was administered to the experimental mice once a day for 2 weeks before ethanol administration. ELG dosage was determined by preliminary studies [28, 36, 37] and the results of our pilot studies. Following the conclusion of the previous ELG administration, the mice were subjected to an overnight fasting period and subsequently gavaged with 56% (v/v) ethanol at a dose of 5 g.kg^−1^ body weight. The normal control mice received an equivalent volume of double-distilled water. After a 16-h interval, the mice were euthanized under the influence of 20% urethane (Sigma, St. Louis, MO) anesthesia, and samples of their cardiac blood and liver tissue were collected. The mouse body weight and liver weight were measured to calculate relevant ratios.

### Serum lipids analysis

To separate the serum from the plasma, the blood samples from mice were centrifuged at 3000 rpm using a freezing centrifuge (5810R, Eppendorf, Eppendorf AG., Hamburg, Germany) for 15 min. The levels of serum triglyceride (TG) and total cholesterol (TC) levels were measured utilizing an automatic biochemical analyzer (Hitachi 7020, Tokyo, Japan).

### Detection of serum liver enzyme levels

The levels of alanine aminotransferase (ALT) and aspartate aminotransferase (AST) in both mouse serum and cell culture supernatant were measured utilizing commercial kits (Jiancheng Bioengineering Institute, Nanjing, China) following the manufacturer’s instructions. After the specified treatment for 24 h, the AML-12 cell culture supernatants were collected from different groups.

### Liver histopathology

The paraffin-embedded liver tissues were sliced into sections measuring 5 μm in thickness. These sections were then subjected to staining with hematoxylin and eosin (H&E), following established protocols. To perform the Oil Red O staining, the tissue sections were first prepared with the OCT and subsequently stained with Oil Red O. Finally, all sections were examined under a light microscope (CX23, Olympus, Olympus Corporation., Tokyo, Japan).

### Determination of liver TG and TC content

The hepatic lipid content was measured in accordance with previous descriptions [38]. Briefly, the liver tissues were weighed and homogenized, followed by lipid extraction with addition of a combination of chloroform and methanol (2:1, v/v) in equal proportions. The resulting lipids-containing layer was collected and dissolved with isopropanol. The hepatic levels of TC and TG were then measured using commercial kits (Jiancheng Bioengineering Institute, Nanjing, China). The weight of liver tissues served as the standard.

### Analysis of relative mRNA expression

The total RNA from mouse tissues and cells was extracted using TRIzol reagent (Invitrogen Life Technologies, MA, USA). The first-strand cDNA was synthesized and subjected to quantitative real-time PCR (qRT-PCR) analysis using SYBR qPCR Master Mix (ABclonal Technology Co., Ltd., Wuhan, China). The ABI StepOnePlus real-time PCR system (Applied Biosystems, USA) was used to analyze the gene expression levels based on the 2-^ΔΔCt^ method. *β-Actin* was used as the internal reference for normalizing all gene expression data. All primer sequences are provided in Additional file 1: Table S3.

### Western blotting

10% SDS-PAGE was used to separate the cells and mouse liver whole protein, and the isolated protein was subsequently transferred onto nitrocellulose membranes. These membranes were blocked with 5% skim milk powder and then incubated with primary antibodies against β-Actin (#3700), phosphorylation of JNK (p-JNK, #4668) (Cell Signaling Technology), AMPK (#AF6423), cleaved Caspase-1 (cle-CASP1, #AF4022), phosphorylation of AMPK (p-AMPK, #AF3423), cleaved Caspase-3 (cle-CASP3, #AF7022) (Affinity Biosciences), C/EBP homologous protein (CHOP, #15204-1-AP), JNK (#15204-1-AP) (Proteintech Group), and NLRP3 (ABclonal Technology, Wuhan, China, #A12694) at a dilution of 1:1000, and incubated overnight at 4 °C. Following this, the membranes were exposed to secondary antibodies labeled with horseradish peroxidase, diluted at a ratio of 1:5000, and incubated for 2 h at room temperature. The protein bands were visualized through chemiluminescence.

### Enzyme-linked immunosorbent assay (ELISA)

A volume of 100 μL of mouse serum or AML cell supernatant was taken for IL-1β measurement by using ELISA Assay Kits (ABclonal Technology), following the instructions provided by the manufacturer.

### Cell counting kit-8 (CCK8) assessment

Cell viability was assessed using the CCK-8 assay following the guidelines provided by the manufacturer (Yeasen, Shanghai, China). In summary, AML-12 cells were plated in 96-well culture plates with a concentration 1 × 10^5^ cells/well in 100 μL of the medium. The plates were then placed in a CO_2_ incubator at 37 °C. Following indicated treatments, 10 μL CCK-8 solution was diluted into each well and incubated for another 1 h at 37 °C. The microplate reader (Synergy^™^4, BioTek, Vermont, USA) was utilized to measure the absorbance values at 450 nm.

### Intracellular oxidative stress assay

The total intracellular levels of ROS were measured using dichlorodihydrofluorescein diacetate (DCFH-DA) (Beyotime, China) according to the manufacturer’s protocol. Briefly, AML-12 cells were rinsed using PBS and subsequently treated with 10 μM DCFH-DA for 30 min at room temperature, while ensuring protection from light. The nuclei were re-stained with 0.5 μg/mL DAPI (Yeasen, Shanghai, China). Following another round of PBS washing, both images and fluorescence intensities were acquired at a wavelength of 488 nm with a Wide Field High Content Screening System (IXM-4, Molecular Devices, USA).

### Immunohistochemistry

For immunohistochemistry analysis, the liver Sections (5 μm) were subjected to immunostaining using a primary antibody against NLRP3 (1:50, ABclonal Technology, #A12694) at 4 °C overnight. Following this, secondary antibodies were incubated. The resulting complex was visualized using the DAB reagent for microscopic examination. Hematoxylin was employed to counterstain the nuclei. Finally, the images were photographed with light microscopy (CX23, Olympus, Olympus Corporation., Tokyo, Japan).

### Immunofluorescence staining

Immunofluorescence was performed on the 5 μm paraffin-sectioned liver samples fixed in formalin. F4/80 primary antibody (1:100, Affinity Biosciences, #DF2789) was incubated at 4 °C overnight on the sections. Afterward, the sections underwent treatment with secondary antibodies labeled with Alexa Fluor 594 (1:600, Yeasen, Shanghai, China) for 60 min at 25 °C. DAPI (0.5 μg.ml^−1^, Yeasen, Shanghai, China) was used to stain the nuclei. The images were photographed with an IXM-4 fluorescent microscope (Molecular Devices, USA).

### Hepatic malondialdehyde and glutathione determination

The liver tissues (50 mg/mouse) were homogenized with 0.5 mL saline using an ultrasonic method in an ice water bath. Subsequently, the homogenates underwent centrifugation at 12,000 rpm for 10 min at 4 °C. The collected supernatants were used to evaluate hepatic lipid peroxidation by quantifying the production of thiobarbituric acid reactive substances (TBARS) through spectrophotometry, with the outcomes reported as the content of malondialdehyde (MDA) content. The levels of hepatic glutathione (GSH) were determined employing a colorimetric technique. Commercial kits (Jiancheng Bioengineering Institute, Nanjing, China) were employed to measure hepatic MDA and GSH contents following the manufacturer’s guidelines.

### Statistical analysis

All data were presented as the means ± SEM. Statistical differences were evaluated using GraphPad 5 (San Diego, CA, US). Significance between two groups with normally distributed data were evaluated by independent two-tailed t-tests. Statistical differences among three or more groups were performed using One-way ANOVA with Bonferroni' s post hoc test. Statistics were considered significant with p < 0.05.

## Results

### Phytochemical characterization of ELG

**Fig. 1:**
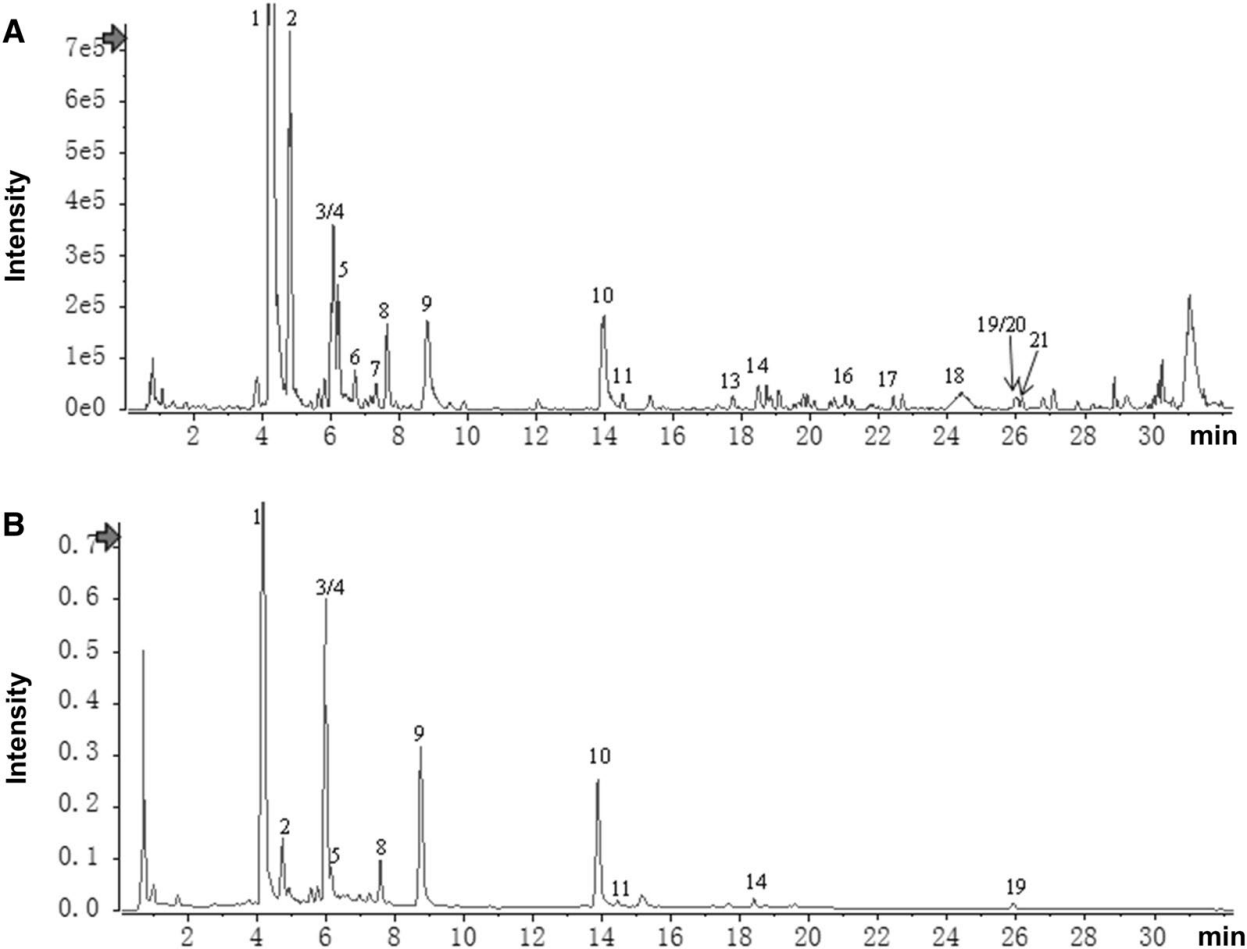
Extract of LanGui tea (ELG) components analysis by Ultra-Performance Liquid Chromatography Quadrupole-Time of Flight Mass Spectrometer (UPLC-Q-TOF–MS). **A** Base peak ion (BPI) chromatogram of ELG in positive ion mode determined by UPLC-HRMS analysis; **B** UPLC-UV chromatogram of ELG (254 nm)

To identify the active components of ELG, ELG powder was subjected to UPLC analysis by UPLC/Q-TOF–MS. A comprehensive of 21 compounds were recognized in ELG using multistage mass spectrometry data (Fig. 1A, B) in conjunction with a natural product high-resolution mass spectrometry database (Additional file 1: Table S2). The chromatogram of UPLC-Q-TOF/MS is shown in Fig. 1. It was observed that the primary active ingredient, referred to as "dihydromyricetin", constitutes a significant proportion of ELG (Fig. 1 and Additional file 1: Table S2). This conclusion is supported by the high-performance liquid chromatography (HPLC) analysis of dihydromyricetin in ELG, which revealed a concentration of 7.23 g dihydromyricetin per 100 g of ELG (Additional file 1: Figure S1). Collectively, this suggests that dihydromyricetin may serve as the principal efficacious component in ELG.

### ELG protects against hepatic steatosis in mice with binge-alcohol-induced ALD

**Fig. 2:**
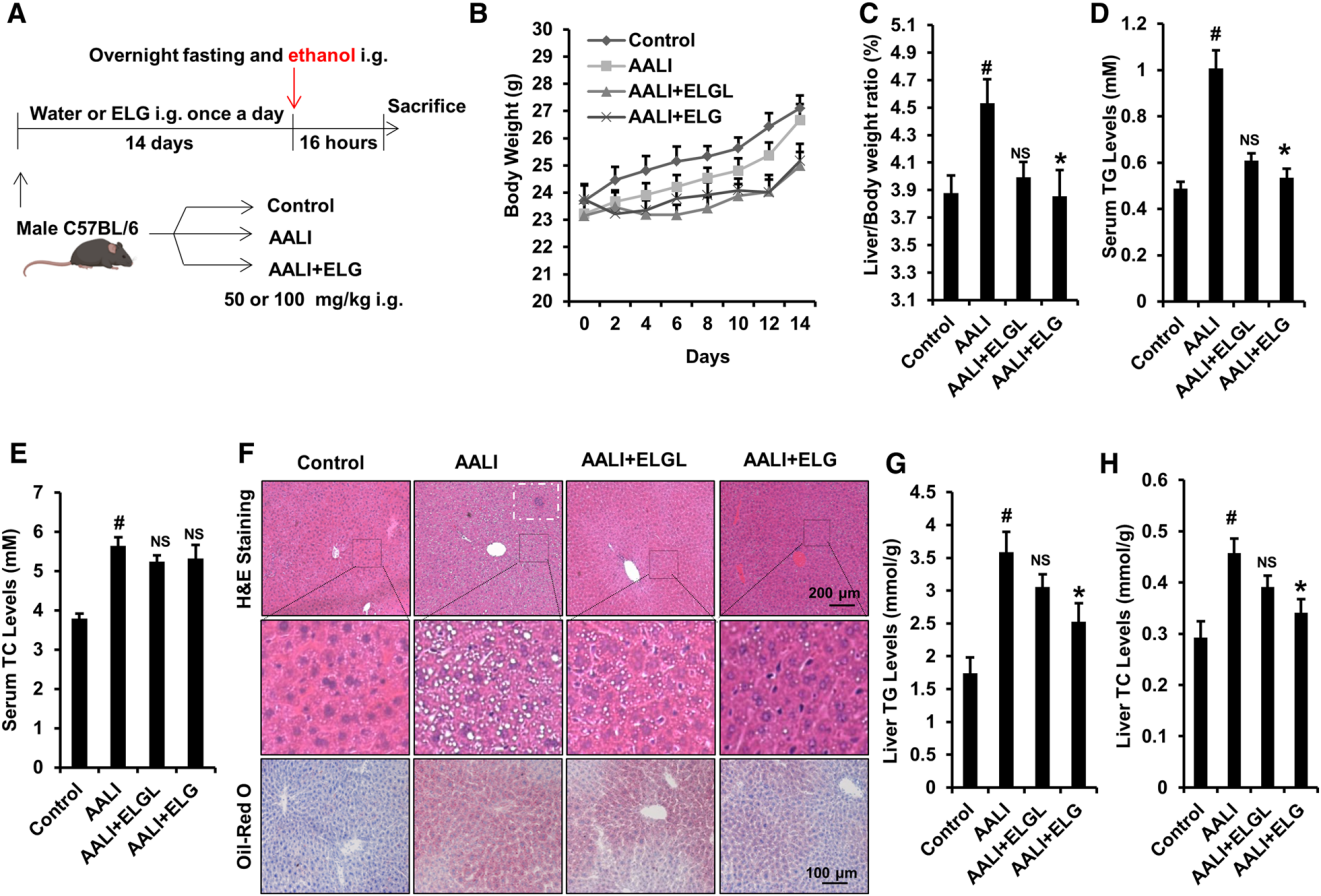
ELG combats acute alcohol-induced fatty liver in mice. **A** Overview of the animal experimental design. **B** Body weight curve of mice. n = 7. **C** Ratio of liver to body weight. n = 7, per group. **D**, **E** Serum TG and TC levels. n = 7. **F** Typical images of mouse liver tissues stained with H&E (original magnification, × 100; Scale bars, 200 µm) and oil red O (original magnification, × 200; Scale bars, 100 µm). n = 7. The white dotted rectangular frame indicates the area of inflammatory cell infiltration. **G**, **H** Mouse TG and TC levels in liver. n = 7. Statistical analysis of all experimental data was performed as means ± SEM. ^#^*p* < 0.05, AALI vs. control group; *p < 0.05, AALI *vs* AALI + ELG group, *NS* not significance

To examine whether ELG can protect mice from binge alcohol-induced AALI, male C57BL/6 J mice were administered component-analyzed ELG once daily for a duration of 2 weeks prior to acute alcohol gavage. According to the results, ELG (50 and 100 mg kg^−1^) did not have a significant impact on mouse body weight (Fig. 2B), but it did reverse the significant liver-to-body weight ratios increase induced by acute alcohol exposure (Fig. 2C). Furthermore, the assay of serum lipid levels demonstrated that binge ethanol administration led to a obviouse increase in serum levels of TG and TC in mice (Fig. 2D, E). Conversely, ELG (100 mg kg^−1^) caused a significant decrease in serum TG levels but did not significantly alter the serum TC levels in mice when compared to the AALI group (Fig. 2D, E). These data indicate that ELG has the potential to mitigate the detrimental effects of binge alcohol consumption on lipid levels in mice. To further verify the beneficial effects of ELG on alleviating binge alcohol-induced fatty liver, liver sections from each experimental group were subjected to pathological analysis using H&E and oil red O staining. As shown in Fig. 2F, ELG effectively inhibited the accumulation of lipid and inflammatory cell infiltration induced by binge ethanol consumption. Furthermore, subsequent determinations of the liver lipid content revealed significantly higher TG and TC levels in mice exposed to alcohol, in contrast to those in the controls (Fig. 2G, H). Interestingly, this increase was not observed in mice treated with ELG (Fig. 2G, H). These results support the histopathological observations. These findings indicate that ELG treatment prevents binge ethanol consumption-induced excessive accumulation of hepatic lipids in mice.

### ELG reduces hepatic inflammation in binge alcohol-induced mice

**Fig. 3:**
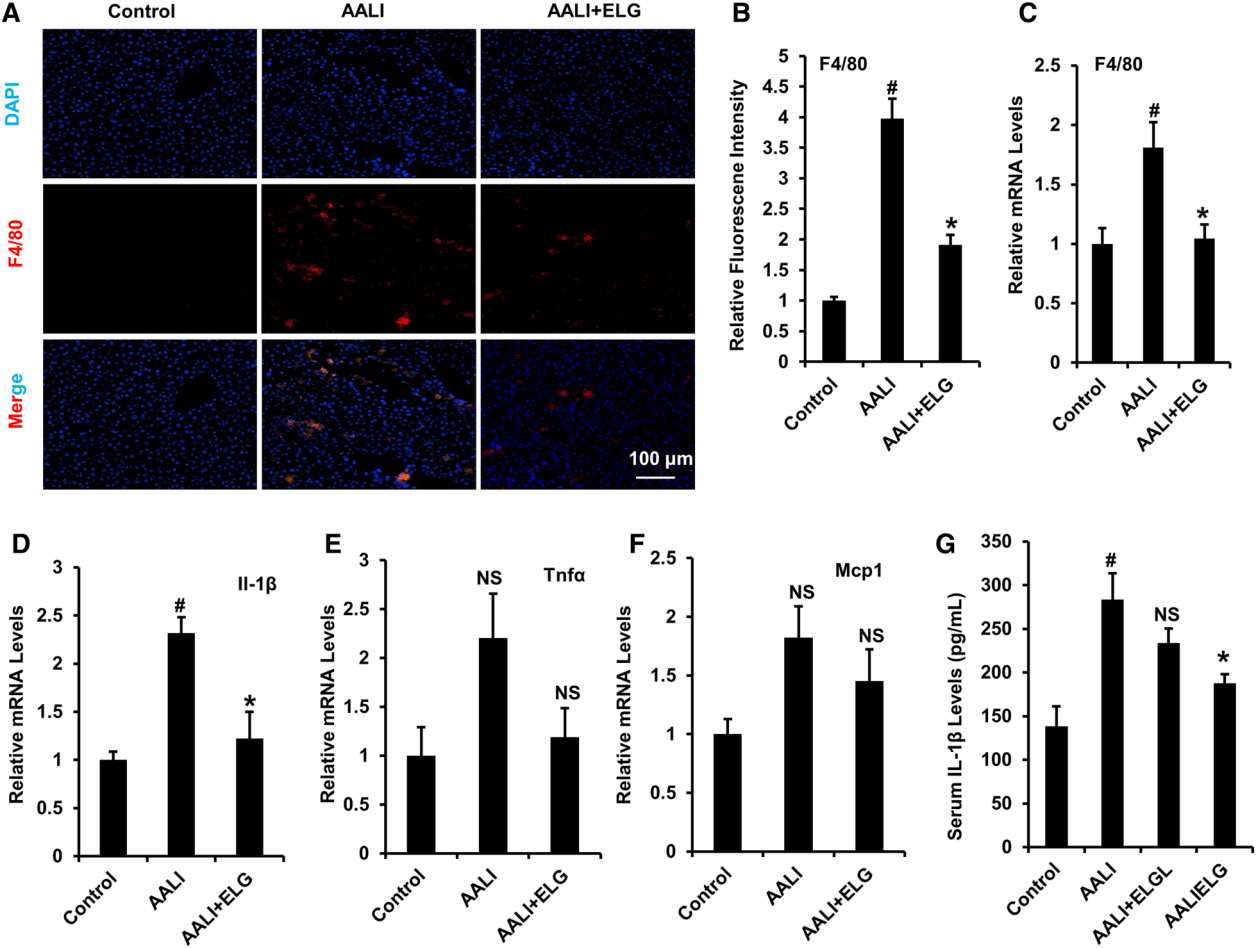
ELG suppresses hepatic inflammation response in acute binge alcohol-treated mice. **A**, **B** Hepatic F4/80 immunofluorescence and relative fluorescence intensity analysis in mice. n = 5. Original magnification, × 200; Scale bars, 100 µm. **C**–**F** Relative mRNA levels of hepatic pro-inflammatory cytokines. n = 5. **G** Serum Il-1β levels. n = 6. Statistical analysis of all experimental data was performed as means ± SEM. ^#^*p* < 0.05, AALI vs. control group; *p < 0.05, AALI vs AALI + ELG group, *NS* not significance

Acute ethanol exposure can cause ASH and even AH [5]. In our animal experimentation, binge alcohol consumption elicited liver inflammation, as indicated by the presence of inflammatory cell aggregation (Fig. 2E) and significantly increased expression of the hepatic macrophage marker F4/80 (Fig. 3A–C) in AALI mice compared to normal control mice. The administration of ELG effectively mitigated these inflammatory alterations (Fig. 2E and Fig. 3A–C). A subsequent analysis of the expression of pro-inflammatory cytokine mRNAs in mouse livers revealed that acute alcohol treatment increased the Il-1β, Tnfα, and Mcp1 expression (Fig. 3D–F), while ELG administration statistically downregulated the increase in the Il-1β mRNA expression (Fig. 3D) in alcohol modeling mice. Meanwhile, the ELG group exhibited decreased Tnfα and Mcp1 mRNA expression compared to the AALI group, although not statistically significant (Fig. 3E, F). In line with the hepatic gene expression findings, AALI mice demonstrated increased serum Il-1β levels in comparison to the normal control mice, and ELG treatment suppressed these elevated levels (Fig. 3G). These results suggested that ELG counteracts hepatic inflammation in alcoholic mice.

### ELG relieves acute ethanol-induced elevation of liver enzymes and impaired hepatocyte viability

**Fig. 4:**
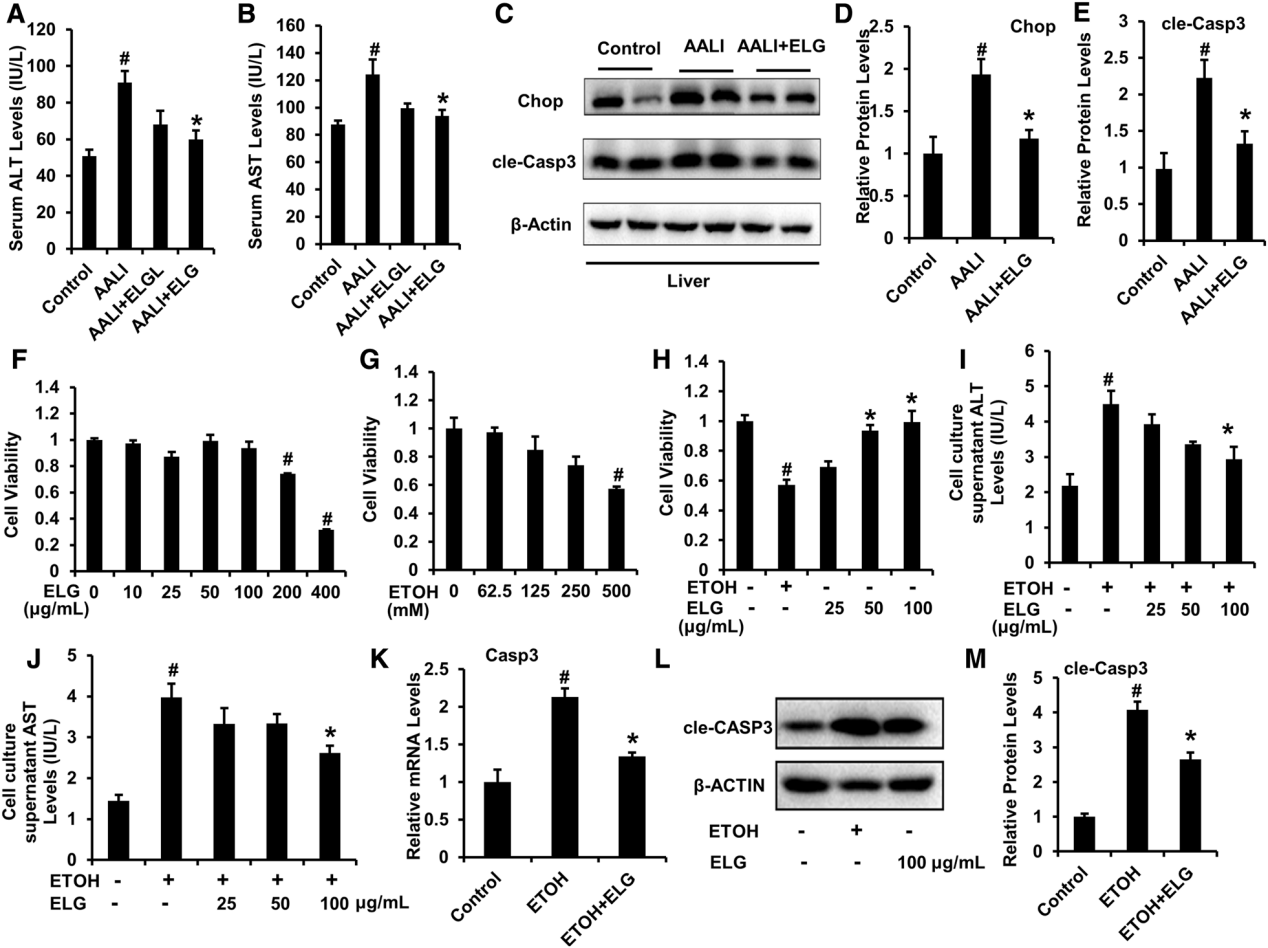
ELG protects against acute ethanol (ETOH)—induced liver enzymes dysfunction and impairment of hepatocyte viability in mice and cell models. **A**, **B** Serum ALT and AST levels. n = 7. **C**–**E** Mouse representative western blot images and relative protein levels in the liver. n = 5. ^#^*p* < 0.05, AALI *vs.* control group; *p < 0.05, AALI *vs* AALI + ELG group. **F**, **G** CCK8 evaluation in AML-12 cells receiving 24-h DMSO, ELG (0, 10, 25, 50, 100, 200 and 400 µg.mL^−1^), or ethanol (0, 62.5, 125, 250 and 500 mM) treatment. n = 3. ^#^*p* < 0.05, control vs ELG or ethanol group. AML-12 cells were incubated with DMSO and ELG (0, 25, 50 and 100 µg.mL^−1^) with or without ethanol (500 mM) for 24 h. **H** CCK8 assay. n = 3. **I**, **J** Cell culture supernatants ALT and AST levels. n = 4. (**K**) The relative mRNA level of Casp3 in AML-12 cells. n = 3. **L**, **M** Representative western blot image and quantitative data of protein expression of cle-Casp3 in AML-12 cells. n = 3. Experimental data were statistically analysed as the means ± SEM. ^#^*p* < 0.05, ethanol *vs.* DMSO control group; *p < 0.05, ethanol *vs* ethanol + ELG group

Subsequently, we assessed whether ELG could improve liver function impairment in acute ethanol-induced mice. Serum ALT and AST levels were elevated in the AALI group compared to the control group (Fig. 4A, B), indicating that alcohol promoted liver injury in mice. However, ELG significantly prevented these increases (Fig. 4A, B), suggesting that ELG-treated mice experienced recovery from liver dysfunction. Binge ethanol consumption-induced hepatic impairment is typically accompanied by apoptotic cell death and excessive ER stress [1]. The increased production of CHOP and cleavage of caspase-3 (cle-CASP3) largely mediate apoptosis and ER stress. As displayed in Figs. 4C–E, binge drinking markedly increased the Chop and cle-Casp3 protein expression, which was largely prevented by ELG treatment. The results suggest that ELG may protect mice from binge-alcohol-induced AALI by reducing hepatic ER stress and apoptosis.

To investigate the potential hepatoprotective effects of ELG against ethanol-induced hepatocyte damage, an in vitro AALI model was employed and treated with ELG at non-cytotoxic concentrations (Fig. 4F). The high ethanol stimulus significantly impaired cell viability, which was effectively reversed by ELG treatment (Fig. 4G, H). Subsequently, the ALT and AST activities in the cell culture supernatant of ethanol-challenged hepatocytes were assessed. ELG notably suppressed ALT and AST production (Fig. 4I, J), indicating the mitigation of cellular damage in ELG-treated hepatocytes. This finding was further corroborated by the analysis of intracellular mRNA expression of Casp3 and protein expression of cle-Casp3 (Fig. 4K–M).

### ELG reduces oxidative stress caused by acute ethanol in liver and cultured hepatocytes

**Fig. 5:**
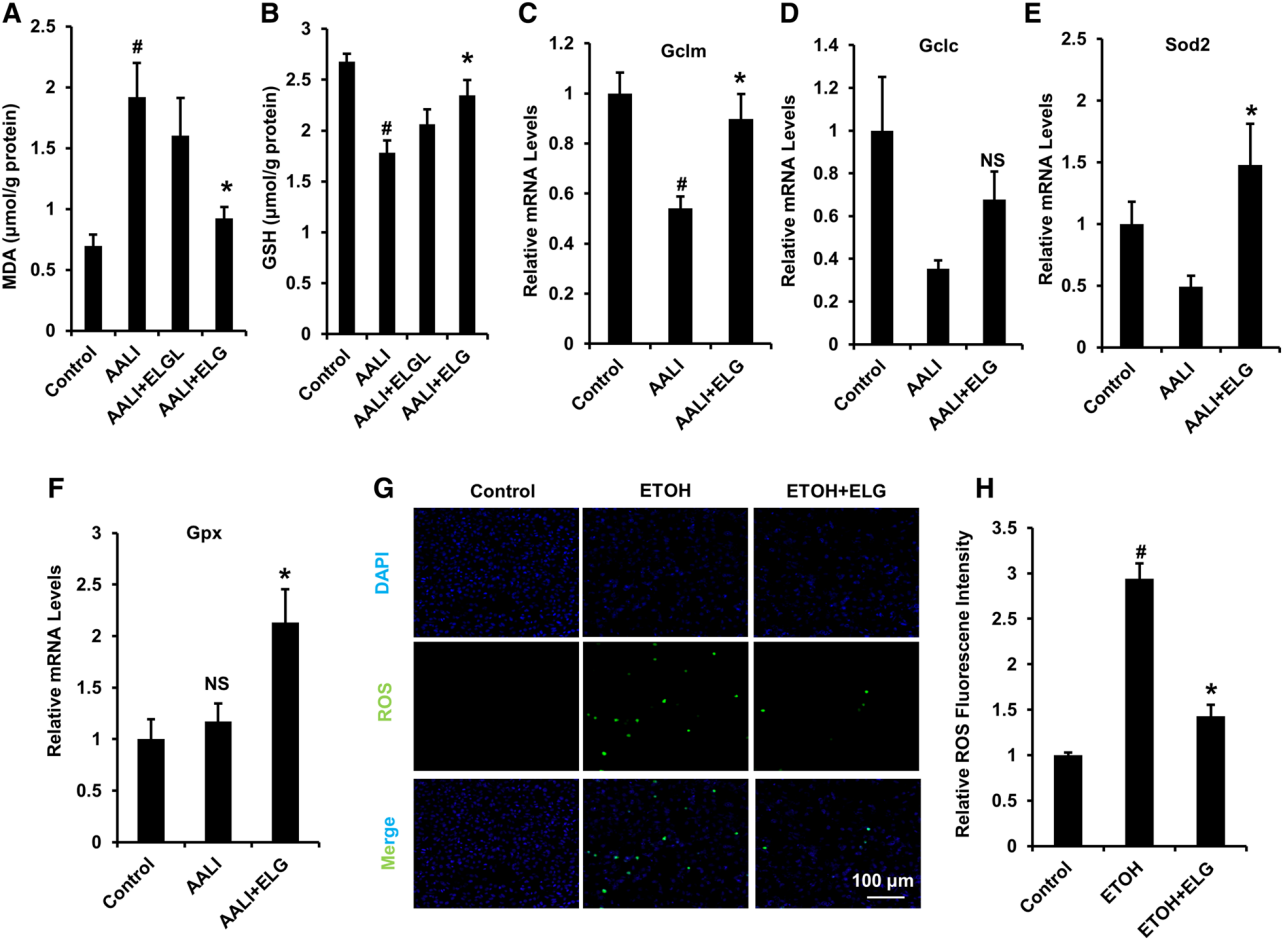
ELG attenuates oxidative stress in liver and AML-12 cells under ethanol challenge. **A**, **B** Analysis of hepatic MDA and GSH contents. n = 6. **C**–**F** mRNA determination of hepatic oxidative stress markers. n = 5. ^#^*p* < 0.05, AALI *vs.* control group; *p < 0.05, AALI vs AALI + ELG group, *NS* not significance. **G**, **H** The representative fluorescent images and relative fluorescent intensity of ROS staining in AML-12 cells receiving 24-h co-incubation of ELG (100 µg.mL^−1^) and ethanol (500 mM). Original magnification, × 200; Scale bars, 100 µm. n = 3. All experimental data were statistically analysed as means ± SEM. ^#^*p* < 0.05, ethanol vs. DMSO control group or *p < 0.05, ethanol *vs* ethanol + ELG group

Hepatic oxidative stress and ROS production lead to hepatocellular damage and contribute to ALD development [39]. While determining the liver MDA and GSH contents, we observed that binge alcohol induced significantly higher liver MDA levels, but distinctly lower liver GSH levels relative to that in the normal controls (Fig. 5). This finding indicated that lipid peroxidation and antioxidant depletion occurred in the mouse liver. In contrast, ELG treatment significantly minimized these alterations in hepatic MDA and GSH contents in AALI mice (Fig. 5A, B). The analysis of gene expression pertaining to hepatic oxidative stress indicators demonstrated a distinct decrease in the relative mRNA expression of hepatic Gclm, Gclc, and Sod2 following acute ethanol treatment (Fig. 5C–E). However, these levels were largely restored upon treatment with ELG (Fig. 5C–E). Additionally, the ELG-treated mice exhibited significantly higher Gpx mRNA expression compared to the mice with AALI (Fig. 5F). These data indicate that ELG attenuated hepatic oxidative stress in binge-alcohol-fed mice. Moreover, in vitro evaluation revealed that ELG effectively inhibited the excessive accumulation of ROS in hepatocytes subjected to ethanol insult (Fig. 5G, H). These results additionally reinforce the the notion that the beneficial effects of ELG in combating ALD are attributed to its anti-oxidative stress activity.

### ELG activates hepatic AMPK-NLRP3 signaling

**Fig. 6:**
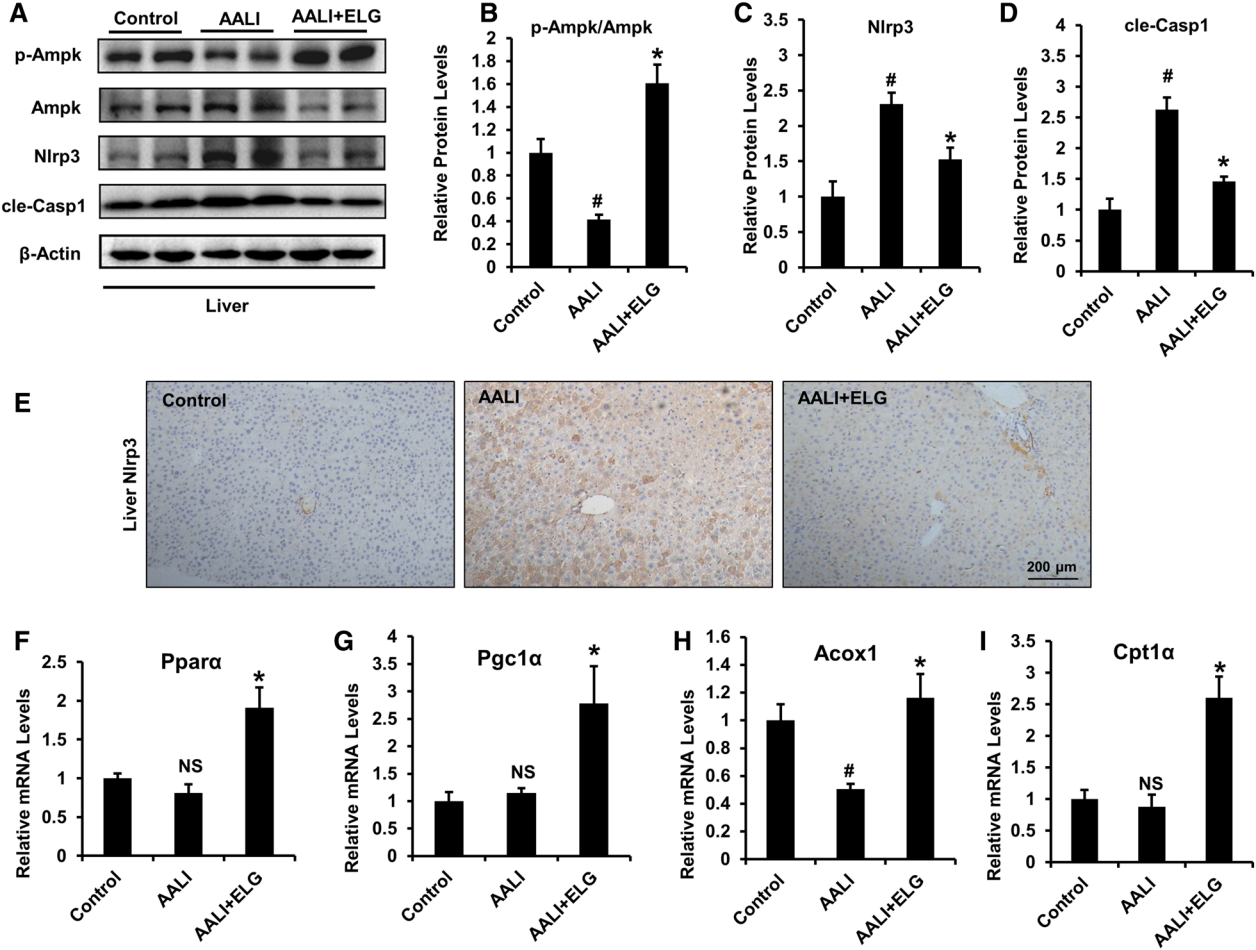
ELG actives hepatic AMPK-NLRP3 signaling in acute binge alcohol-fed mice. Representative western blot image (**A**) and relative protein expression of p-Ampk/Ampk (**B**), Nlrp3/β-Actin (**C**), and cle-Casp1/β-Actin (**D**) in the liver of mice from control, AALI and AALI + ELG group. n = 5, per group. **E** Hepatic immunohistochemical analysis of Nlrp3. n = 5, per group. Original magnification, × 100; Scale bars, 200 µm. **F**–**I** Relative mRNA levels of FAO-involved hepatic AMPK downstream genes. n = 5. Statistical analysis of all experimental data was performed as means ± SEM. ^#^*p* < 0.05, AALI *vs.* control group; *p < 0.05, AALI *vs* AALI + ELG group, *NS* not significance

Given the observed similarity between the previously mentioned anti-ALD activity of ELG and the regulatory function of AMPK-NLRP3 signaling in ALD control, we investigated the potential association between hepatic AMPK-NLRP3 signaling and the underlying action mechanisms of action of ELG. Binge drinking was found to significantly decrease AMPK phosphorylation (Fig. 6A, B), while simultaneously increasing the hepatic expressions of Nlrp3 and cle-Casp1 proteins (Fig. 6 A, C and D), indicating inhibition of AMPK signaling and activation of NLRP3 signaling in the mouse liver. Nevertheless, ELG treatment efficiently rescued these changes in the protein levels (Fig. 6A–D). In addition, hepatic immunohistochemical analysis of NLRP3 supported that ELG relieved alcohol-induced increase in the protein expression of NLRP3 in situ tissues (Fig. 6E). Furthermore, ELG treatment exhibited a significant downregulation of hepatic Il-1β expression (Fig. 3D) and serum Il-1β (Fig. 3G) levels in ALD mice, while also increasing the hepatic mRNA expression of FAO-associated AMPK downstream genes Pparα, Pgc1α, Acox1, and Cpt1α (Fig. 6F–I). These results implied that ELG could modulate hepatic AMPK-NLRP3 signaling.

**Fig. 7:**
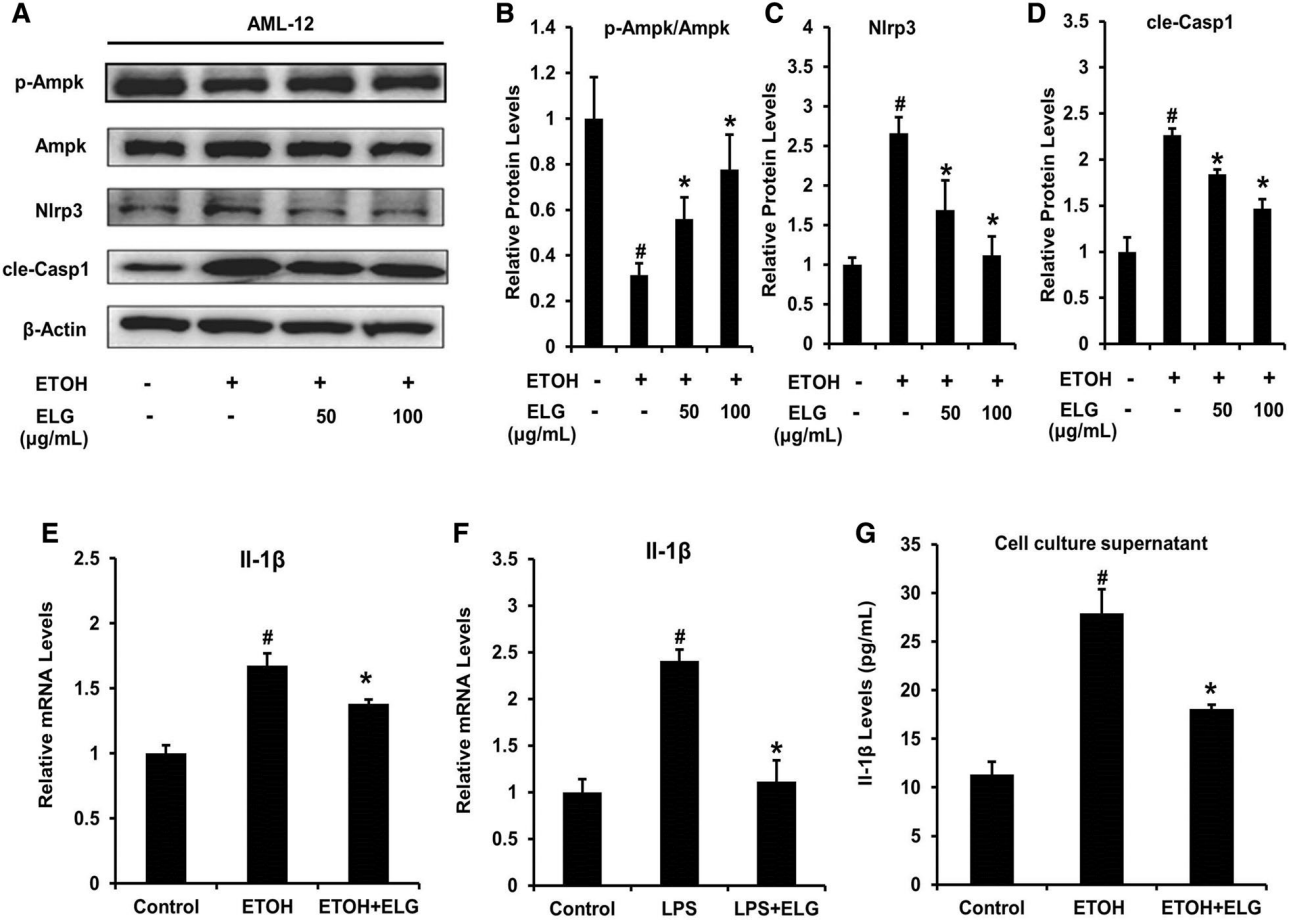
ELG regulates AMPK-NLRP3 signaling in mouse hepatocytes. AML-12 cells were treated by DMSO and ELG (0, 50 and 100 µg.mL^−1^) with or without ethanol (500 mM) for 24 h. Representative western blot image (**A**) and relative protein levels of genes (**B**–**D**) in AMPK-NlRP3 signaling. n = 3, per group. **E**, **F** AML-12 cells receiving culture of DMSO, ethanol (500 mM) or LPS (100 ng.mL^−1^) and treated in the presence or absence of ELG (100 µg.mL^−1^) were used for subsequent analysis of relative mRNA levels of Il-1β. n = 3, per group. **G** Cell supernatant Il-1β concentration in AML-12 with ethanol (500 mM) stimulation and ELG (100 µg.mL^−1^) treatment. n = 3, per group. Data were statistically analysed as means ± SEM. ^#^*p* < 0.05, as compared with control group; *p < 0.05, ELG treatment *vs* ethanol or LPS group

In accordance with the results obtained from the mouse liver, we confirmed that ELG effectively activated AMPK-NLRP3 signaling in hepatocytes. This is supported by the observed improvement in AMPK phosphorylation upon ELG treatment in vitro (Fig. 7A, B), as well as the inhibition of the Nlrp3 and cle-Casp1 protein levels in hepatocytes under ethanol-induced conditions (Fig. 7A, C and D). Additionally, our findings demonstrated that ELG disrupted the mRNA expression (Fig. 7E, F) and production of Il-1β (Fig. 7G) in cultured mouse hepatocytes stimulated with ethanol or LPS. These data imply that AMPK-NLRP3 signaling activation in hepatocytes may play a role in the protective impact of ELG against binge drinking-induced AALI.

### AMPK inactivation curbs ELG inhibiting ethanol-triggered NLRP3 signaling and damage in hepatocytes

**Fig. 8:**
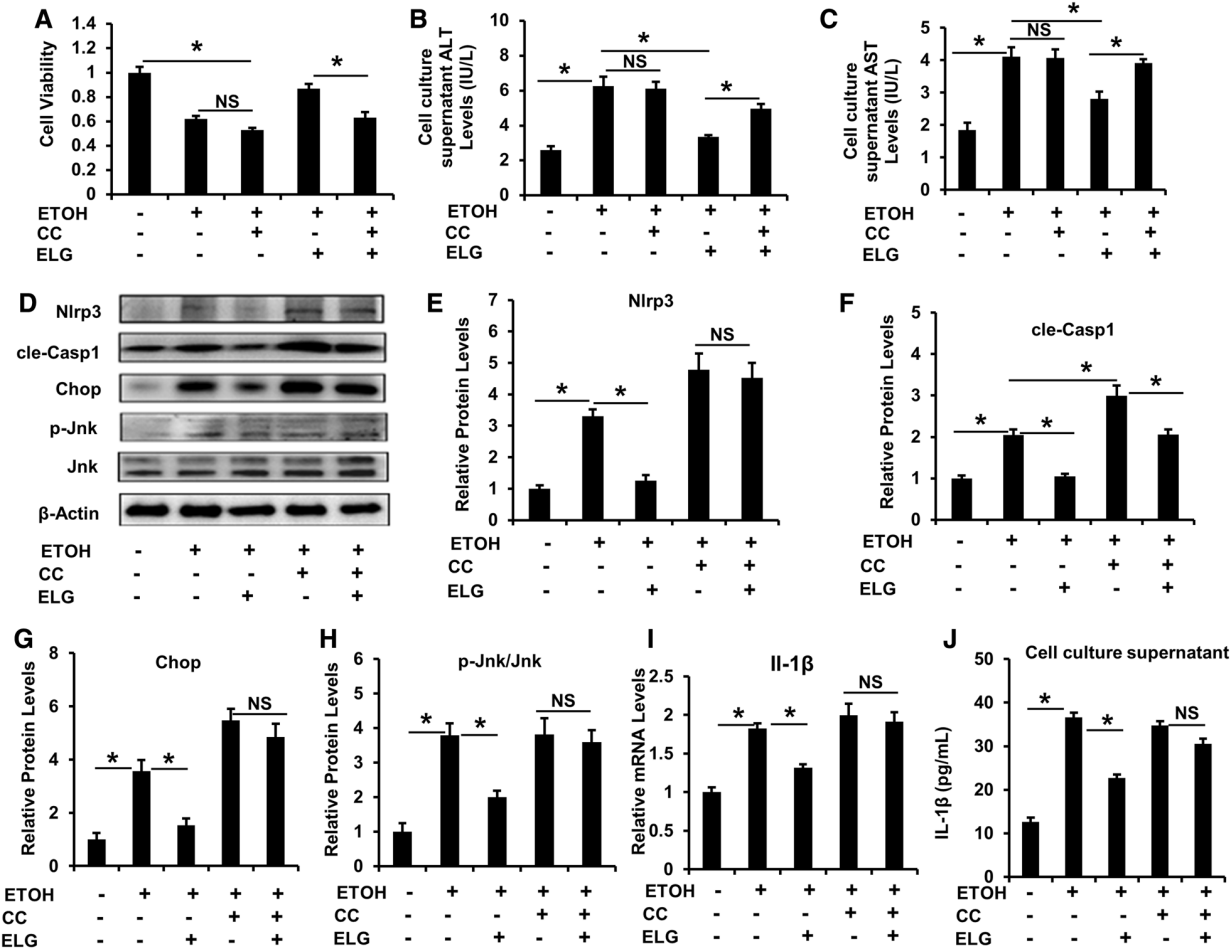
AMPK inactivation blunts the inhibitory effects of ELG on NLRP3 signaling and impairment in mouse hepatocytes. AML-12 cells insulted by ethanol (500 mM) were treated by DMSO and ELG (100 µg.mL^−1^) with or without CC (10 µM) for 24 h. **A** Cell viability assessment. n = 3. **B**, **C** ALT and AST levels in cell culture supernatants. n = 3. Representative western blot image (**D**) and relative protein expression of Nlrp3 (**E**), cle-Casp1 (**F**), Chop (**G**) and p-Jnk/Jnk (**H**) were displayed. n = 3. **I** Determination of relative mRNA expression of Il-1β. n = 3. **J** The cell supernatant Il-1β level. n = 4, per group. Data were statistically analysed as means ± SEM. *p < 0.05, as compared with ethanol group or ethanol + ELG + CC group, *NS* not significance

To verify whether ELG-induced AMPK activation mediates the inhibition of NLRP3 signaling and hepatocyte injury under ethanol-induced conditions. CC, a selective AMPK inhibitor, was used to hinder AMPK signaling in vitro. ELG exhibited a significant protective effect on hepatocyte viability (Fig. 8A) and ALT and AST production in the cell culture supernatants (Fig. 8B, C) following ethanol stimulation. However, this protective effect was diminished when co-cultured with CC (Fig. 8A–C). Moreover, CC treatment exacerbated the expression of Nlrp3, cle-Casp1, and Chop protein in hepatocytes induced by ethanol (Fig. 8D–G), while it nearly nullified the suppressive impact of ELG on ethanol-induced increase in Nlrp3, cle-Casp1, Chop, and the p-Jnk/Jnk protein expression (Fig. 8D–H). Additionally, although the combination of CC and ethanol did not synergistically increase the expression of Il-1β mRNA (Fig. 8I) and protein release (Fig. 8J) in cultured hepatocytes, CC restrained the inhibitory effect of ELG on intracellular Il-1β mRNA expression and production under ethanol incubation conditions (Fig. 8I, J). These results suggest that ELG defends against ethanol treatment-induced hepatocyte injury and the overactivation of NLRP3 inflammasome signaling through AMPK activation.

## Discussion

**Fig. 9:**
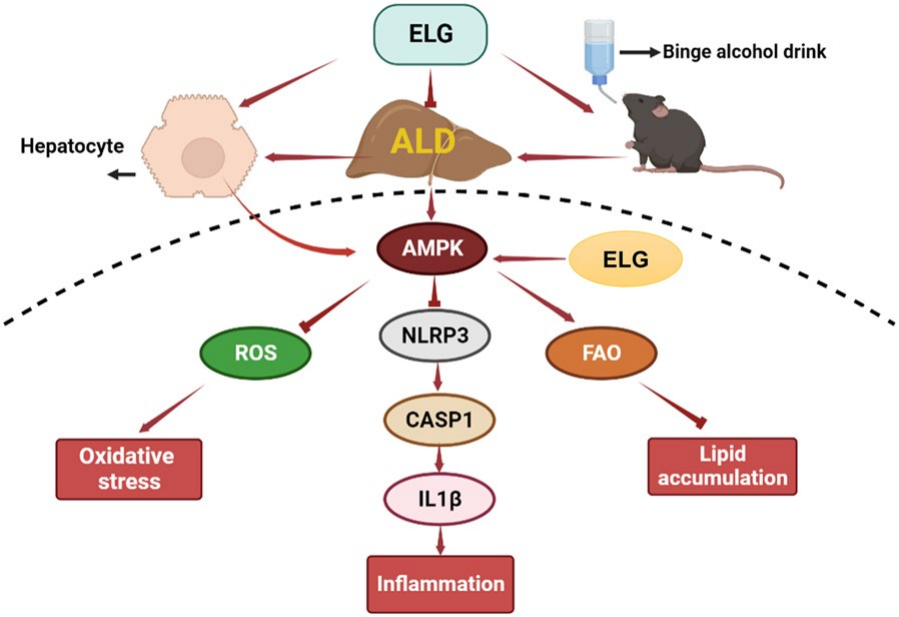
Proposed mechanisms of ELG in protecting acute binge alcohol-induced fatty liver. The main effect of ELG in protecting against acute alcohol-induced fatty liver may through activation of AMPK in hepatocytes, thus inhibiting NLRP3/Caspase1/Il-1β signaling. Consequently, ELG inhibits inflammation and oxidative stress injury in hepatocytes and promots hepatic fatty acid oxidation

Traditional herbal medicines offer significant potential in ALD prevention and treatment owing to their effectiveness and low toxicity. The role of LanGui tea as a medicinal and functional tea formula in ALD management remains obscure. In this study, ELG attenuated binge alcohol-induced hepatic inflammatory response, lipid accumulation, and liver injury in mice. Mechanistically, ELG activated AMPK signaling, while simultaneously repressing NLRP3 inflammasome signaling in the mouse liver. Additionally, ELG was observed to enhance hepatic FAO and alleviate oxidative stress and ER stress (Fig. 9). Our findings indicate that ELG may be beneficial in controlling ALD.

LanGui tea comprises *G. pentaphyllum*, cinnamon, and vine tea. These components possess potential applications in medicine and food due to their diverse pharmacological activities including anti-inflammatory, antioxidation, antifibrosis, antiobesity, hepatoprotective, and lipid-lowering effects [27–29]. Notably, *G. pentaphyllum* and gypenosides have shown promise in alleviating hepatic steatosis, mitochondrial impairment, and inflammatory response, ultimately improving liver dysfunction, particularly in cases of fatty liver [28, 40]. Furthermore, cinnamon has demonstrated therapeutic benefits by reducing hyperlipidemia, liver enzymes, insulin resistance, and hepatic steatosis in both NAFLD patients and animal models [27, 41]. Intriguingly, a recent study has suggested that the vine tea extract and ampelopsin may have the ability to decrease the levels of serum ALT and AST, as well as hepatic TG levels in alcohol-fed mice [36]. These studies strongly suggest the potential of ELG in improving ALD.

Acute alcohol exposure can also mimic the effects of early stages of alcoholic liver injury, including hepatic steatosis, oxidative stress, ER stress, and cellular damage [42]. Furthermore, binge drinking can accelerate fatty liver progression toward hepatitis and even liver cancer [5]. In this study, we found that ELG substantially mitigated liver lipids levels and corrected dysfunctions in serum TG and liver enzyme metabolism in binge alcohol-modeled mice. In addition, ELG substantially mitigated acute binge-alcohol-induced hepatic inflammatory response, as manifested by the prevention of hepatic inflammatory cell accumulation and suppression of proinflammatory cytokine Il-1β expression and generation in both liver and serum. Enhanced ER stress accelerated liver steatosis, inflammation, and apoptosis by activating CHOP and caspase [1]. ELG significantly reduced the hepatic protein levels of Chop and cle-Casp3 protein in AALI controls, implying that ELG ameliorated ER stress and liver injury at the molecular level. These protective effects of ELG align with previously reported pharmacological effects of ELG components in other animal models [27, 28, 32, 41]. In support of the in vivo efficacy of ELG, our in vitro findings further revealed that ELG could effectively attenuate injury in hepatocytes because of its ability to prevent cell viability impairment and the abnormal generation of liver enzymes. ELG could also reverse ethanol-induced cle-Casp3 expression. Thus, our results suggest that ELG may retard the progress of acute alcohol-induced ALD partially through its protective role in hepatocytes.

Oxidative stress is a key mechanism driving ALD by mediating lipid peroxidation, steatosis, and inflammation, ultimately inducing hepatocyte damage through apoptosis, necrosis, and necroptosis [39]. Hepatocytes exposed to ethanol exhibit heightened susceptibility to oxidative stress, resulting in apoptosis through the induction of ROS generation and accumulation, which is accompanied by the production of the toxic peroxidation product MDA and depletion of GSH, the major cellular antioxidant [39]. ROS can damage mitochondrial fatty acid β-oxidation, resulting in the acceleration of fatty liver development [43]. *G. pentaphyllum* and its active components, such as *gynostemma glycosides* and polysaccharides, exhibit substantial antioxidant activity in different tissues of multiple diseases, including increased superoxide dismutase (SOD) and glutathione peroxidase activity and decreased MDA content [28]. Cinnamon and its functional ingredients protect against liver damage by reducing oxidative stress [44, 45]. Dihydromyricetin corrects dysfunctions in myocardial cells and human umbilical vein endothelial cells through oxidative stress inhibition, which is manifested as the reduction of ROS generation and MDA content, but enhanced SOD activity [46]. Similar to the results of previous studies, ELG prevented the binge drinking-induced increase in the MDA content and decrease in the GSH levels in the mouse liver and inhibited ethanol-mediated ROS production in hepatocytes. These findings suggest that ELG mitigates the hepatic production of toxic substances while promoting antioxidant synthesis, potentially attributed to the scavenging of ROS in ELG-treated hepatocytes. ELG also upregulated the hepatic mRNA levels of Gclm, Gclc, Sod2, and Gpx. Once induced, these genes facilitate glutathione synthesis, ROS scavenging, and glutathione reduction of organic hydrogen peroxide, thereby protecting cells from oxidative damage. This finding provides further arguments in favor of the antioxidant role of ELG in ALD treatment.

Alcohol exposure can inhibit the AMPK activity both in vivo and in vitro [47]. AMPK activation can reduce lipid de novo synthesis, ROS generation, ER stress, and inflammation, while promoting FAO and energy metabolism in liver [47]. The activation of NLRP3 inflammasome and IL-1β maturation also contribute to ALD progress. Ethanol exposure results in the activation of the NLRP3 inflammasome and caspase 1, thereby enhancing the serum IL-1β levels [48]. IL-1β promotes inflammation and liver injury in ALD mice, thereby mediating activation of profibrogenic cytokines, damage to hepatocytes, and leukocyte infiltration [16]. Pharmacological blockage of IL-1β signaling improves alcohol-induced liver inflammation, steatosis, and injury [49]. Moreover, deficiency of NLRP3 alleviates liver steatosis, inflammation, and injury [50]. Accumulating evidence has indicated that the activation of AMPK-mediated suppression of the NLRP3 signaling occurs in various tissues and is implicated in the development of different diseases, including ALD [20, 23].

Notably, *G. pentaphyllum*, cinnamon, and vine tea can improve metabolic dysfunctions by inducing AMPK signaling [28, 32, 37]. Furthermore, *G. pentaphyllum* polysaccharides inhibit hepatic expression of the NLRP3 and IL-1β in mice, thereby ameliorating NASH [51]. Gypenosides alleviate diabetic cardiomyopathy by preventing the activation of NLRP3 inflammasome [52]. Dihydromyricetin reduces methotrexate-induced hepatotoxicity by inhibiting the hepatic NLRP3/caspase 1 signaling [53]. Therefore, given the significance of AMPK and NLRP3 signaling in the regulation of ALD and the alignment of previous findings on the components of LanGui tea with our current findings on the beneficial effects of ELG in resolving liver steatosis, oxidative stress, inflammation, and damage, and regulating AMPK and NLRP3 signaling, it is hypothesized that ELG prevents binge drinking-induced ALD by modulating hepatic AMPK-NLRP3 signaling. AMPK activation-mediated repression of NLRP3 inflammasome mainly involves multiple mechanisms including decreasing cellular ROS levels, promoting autophagy, alleviating ER stress, and inducing SIRT1-mediated deacetylation, thereby reducing the expression and activity of NLRP3 inflammasome [19]. Here, ELG notably enhanced hepatic phosphorylation of Ampk and its downstream genes Pparα, Pgc1α, Acox1, and Cpt1α in alcohol-treated mice. These genes are essential for promoting fatty acid β-oxidation. Meanwhile, ELG counteracted alcohol-induced hepatic oxidative stress and ER stress in mice. These findings suggest that ELG activates hepatic AMPK and the anti-ALD effect of ELG involves AMPK-related regulation of fatty acid β-oxidation, oxidative stress, and ER stress. On the other hand, in addition to preventing the mRNA expression and release of NLRP3 signaling downstream pro-inflammatory molecules Il-1β, ELG treatment robustly reduced the hepatic protein expression of Nlrp3 and cle-Casp1 in alcohol-modeled mice. Additionally, we found that ELG diminished ROS generation in alcohol-induced hepatocytes. Thus, it is suggested that ELG may inhibit hepatic NLRP3 signaling in acute alcohol-induced mice, possibly through the stimulation of AMPK, which causes the removal of ROS and ER stress. In support, we found that ELG replicated its in vivo pharmacological effects in alcohol-induced hepatocytes, which encompassed the preservation of cell vitality and improvement in liver enzyme dysfunction. The actions encompassed modulation in Ampk phosphorylation, alteration in the expression of crucial Nlrp3 signaling components and pertinent cell damage indicators Chop and p-Jnk, and reduction in Il-1β production. However, such alternations were dramatically impaired by pharmacologically inhibiting AMPK activity using CC—a selective AMPK inhibitor. This discovery suggests that the mitigation of AALI induced by ELG involves the suppression of NLRP3 signaling in hepatocytes mediated by AMPK activation, which may provide a partial explanation for the underlying mechanism of action of LanGui tea in treating ALD.

As a safe herbal formula tea, LanGui tea displays long been used for health care and liver protection in the folk. Thus, LanGui tea has a strong potential in the clinical treatment of ALD and related liver injury. However, its clinical relevance warrants further investigation. Our current study has some limitations. We have discovered that ELG contains a ubstantial quantity of dihydromyricetin, which has been documented to possess a defensive impact against ALD [54]. However, additional exploration is warranted to examine the anti-ALD effects exerted by other constituents of ELG. Our present study focused on the preventive effects of LanGui tea in acute binge alcohol-induced mice. However, the therapeutic roles of LanGui tea in acute and chronic ALD warrants further investigation. In addition, Our investigation has established that ELG hinders NLRP3 signaling by repressing the crucial NLRP3 inflammasome components expression through the activation of AMPK. However, further comprehensive mechanistic investigations are required to investigate, for example, whether ELG affects the assembly of NLRP3 inflammasome multiprotein complexes. Although NLRP3 functions in immune cells have gained extensive attention, NLRP3 signaling activation also contributes to hepatocyte pyroptosis, inflammation, and liver damage [55]. We demonstrated that the in vivo hepatoprotective effect of ELG may be the result of ELD-mediated AMPK-NLRP3 signaling activation in hepatocytes. This finding hints at the potential existence of analogous activities of ELG in other liver cells including monocytes and macrophages, which may mediate the anti-ALD actions of ELG and require clarification.

## Conclusions

In summary, we demonstrated, for the first time, that LanGui tea is a natural modulator of AMPK-NLRP3 signaling. This herbal tea may play a role in ameliorating acute alcohol-induced ALD. The potential mechanism of action of ELG may involve the inhibition of NLRP3 signaling in hepatocytes through AMPK activation. Our results offer the potential pharmacological mechanisms of LanGui tea in counteracting AALI. These discoveries suggest that LanGui tea shows potential as a beneficial treatment for ALD management.

### Supplementary Information

**Additional file 1: Figure S1.** High Performance Liquid Chromatography (HPLC) analysis of dihydromyricetin in ELG. (A) HPLC analysis of dihydromyricetin standard product. (B) HPLC analysis of dihydromyricetin in ELG. **Table S1.** The gradient elution program of the mobile phase. **Table S2.** Identification results of main components of ELG. **Table S3.** Sequences of the mouse primers used in real-time PCR.

## Data Availability

Data in the current study will are available on reasonable request.

## Abbreviations

AALI: Acute alcoholic liver injury
ALD: Alcoholic liver disease
AMPK: Adenosine monophosphate-(AMP)-activated protein kinase
CASP: Caspase
CPT1: Carnitine palmitoyltransferase 1
DMSO: Dimethyl sulfoxide
ELG: Extract of LanGui tea
ER: Endoplasmic reticulum
ETOH: Ethanol
IL-1β: Interleukin 1β
LPS: Lipopolysaccharides
MDA: Malondialdehyde
NLRP3: NLR family pyrin domain containing 3
