# Supplementary material for: LanGui tea, an herbal medicine formula, protects against binge alcohol-induced acute liver injury by activating AMPK-NLRP3 signaling
Source: Chin Med. 2024 Mar 4;19:41. doi: 10.1186/s13020-024-00906-0 (PMC10910869; doi:10.1186/s13020-024-00906-0)
Supplement: Supplementary file 1 — Additional file 1: Figure S1. High Performance Liquid Chromatography (HPLC) analysis of dihydromyricetin in ELG. (A) HPLC analysis of dihydromyricetin standard product. (B) HPLC analysis of dihydromyricetin in ELG. Table S1. The gradient elution program of the mobile phase. Table S2. Identification results of main components of ELG. Table S3. Sequences of the mouse primers used in real-time PCR. [file 13020_2024_906_MOESM1_ESM.docx]

**Additional file**

**LanGui tea, an herbal medicine formula, protects against binge alcohol-induced acute liver injury by activating AMPK-NLRP3 signaling**

***Ming Gu^1^**#*, Yu-jun Chen^1^*#*, Ya-ru Feng^2^ and Zhi-peng Tang^1^***

1 Institute of Digestive Disease, Longhua Hospital, Shanghai University of Traditional Chinese Medicine, Shanghai 200032, China

2 The Third People's Hospital Affiliated to Nantong University, Nantong 226006, Jiangsu Province, China

# These authors contributed equally to this work

*Correspondence: Ming Gu, gu2006122116@126.com


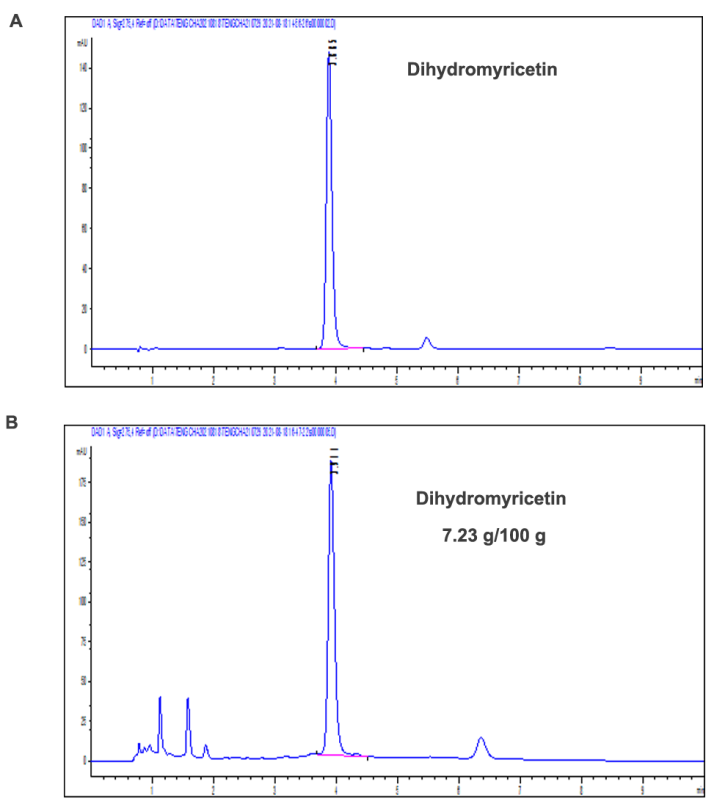


**Figure S1.** **High Performance Liquid Chromatography (HPLC) analysis of dihydromyricetin in ELG**. (**A**) HPLC analysis of dihydromyricetin standard product. (**B**) HPLC analysis of dihydromyricetin in ELG.

**Table S1. The gradient elution program of the mobile phase**

| Time (min) | A% | B% |
| --- | --- | --- |
| 0-5 | 10-20 | 90-80 |
| 5-9 | 20 | 80 |
| 9-16 | 20-30 | 80-70 |
| 16-26 | 30-45 | 70-55 |
| 26-32 | 45-95 | 55-5 |
| 32-36 | 95 | 5 |
| 36-37 | 95-10 | 5-90 |
| 37-40 | 10 | 90 |

**Table S2. Identification results of main components of ELG**

| NO | | Retention time（min） | | Adducts | Measured (M/Z) | Expected  (M/Z) | Error  (ppm) | Formula | Molecular weight | Phytochemical name | MS/MS spectra | |
| --- | --- | --- | --- | --- | --- | --- | --- | --- | --- | --- | --- | --- |
| 1 | 4.33 | | [M-H]^-^ | | 319.0461 | 319.0459 | 0.5 | C_15_H_12_O_8_ | 320.05 | Dihydromyricetin | | 193.0138;175.0037;151.0038;125.0244 |
| 2 | 4.79 | | [M+H]^+^ | | 321.0605 | 321.0605 | 0.0 | C_15_H_12_O_8_ | 320.05 | (2R,3S) -5,7,3',4',5'-  pentahydroxyflavanonol | | 275.0542;247.0593195.0279;153.0172; 149.0225;139.0383 |
| 3 | 6.01 | | [M-H]^-^ | | 609.1465 | 609.1461 | 0.6 | C_27_H_30_O_16_ | 610.15 | Rutin | | 609.1471;300.0282;271.0268 |
| 4 | 6.08 | | [M-H]^-^ | | 463.0880 | 463.0882 | -0.4 | C_21_H_20_O_12_ | 464.10 | Myricitrin | | 463.0891;316.0214;287.0188;271.0240 |
| 5 | 6.22 | | [M-H]^-^ | | 637.0840 | 637.0835 | 0.8 | C_30_H_22_O_16_ | 638.09 | Dimer dihydromyricetin or isomer | | 511.0522;493.0421;449.0522;385.0203 |
| 6 | 6.71 | | [M-H]^-^ | | 637.0839 | 637.0835 | 0.6 | C_30_H_22_O_16_ | 638.09 | Dimer dihydromyricetin or isomer | | 619.0843;511.0593;493.0486;449.0576;385.0249 |
| 7 | 7.30 | | [M+H]^+^ | | 639.0985 | 639.0981 | 0.7 | C_30_H_22_O_16_ | 638.09 | Dimer dihydromyricetin or isomer | | 603.0760;469.0749;301.0340;195.0282;153.0175 |
| 8 | 7.65 | | [M-H]^-^ | | 447.0936 | 447.0933 | 0.7 | C_21_H_20_O_11_ | 448.10 | Quercitrin | | 447.0916;300.0261;271.0244;178.9977;151.0029 |
| 9 | 8.83 | | [M-H]^-^ | | 317.0303 | 317.0303 | 0.0 | C_15_H_10_O_8_ | 318.04 | Myricetin | | 317.0308;178.9997;151.0252;137.0252;109.0299 |
| 10 | 13.95 | | [M-H]^-^ | | 301.0355 | 301.0354 | 0.4 | C_15_H_10_O_7_ | 302.04 | Quercetin | | 301.0342;178.9984;151.0034;121.0291;107.0134 |
| 11 | 14.52 | | [M-H]^-^ | | 637.1771 | 637.1774 | -0.5 | C_29_H_34_O_16_ | 638.18 | Ombuoside | | 637.1817;329.0673;314.0430;299.0176 |
| 12 | 15.22 | | [M+H]^+^ | | 133.0648 | 133.0648 | 0.1 | C_9_H_8_O | 132.06 | Cinnamaldehyde | | 133.0677;115.0557;103.0562;77.0393 |
| 13 | 17.73 | | [M-H]^-^ | | 285.0405 | 285.0405 | 0.1 | C_15_H_10_O_6_ | 286.05 | Kaempferol | | 285.0399;239.0338;211.0383;185.0618;143.0503 |
| 14 | 18.48 | | [M-H]^-^ | | 315.0509 | 315.0510 | -0.4 | C_16_H_12_O_7_ | 316.06 | Isorhamnetin | | 315.0495;300.0262;255.0303;151.0034 |
| 15 | 18.85 | | [M+H]^+^ | | 163.0752 | 163.0754 | -1.0 | C_10_H_10_O_2_ | 162.07 | Methyl cinnamate | | 145.0652;131.0477;115.0531;105.0699;91.0536;77.0378 |
| 16 | 21.00 | | [M-H]^-^ | | 1107.597 | 1107.5957 | 1.5 | C_54_H_92_O_23_ | 1108.60 | Gypenoside XIX | | 1061.5868;899.5372;737.4898 |
| 17 | 22.41 | | [M+FA-H]^-^ | | 991.5484 | 991.5483 | 0.1 | C_48_H_82_O_18_ | 946.55 | Ginsenoside Re | | 945.5455;799.4885;637.4350;475.3810 |
| 18 | 24.43 | | [M+FA-H]^-^ | | 959.5227 | 959.5221 | 0.6 | C_47_H_78_O_17_ | 914.52 | Phanoside | | 959.5270;913.5221;751.4697;667.41097 |
| 19 | 25.98 | | [M-H]^-^ | | 329.0664 | 329.0667 | -0.8 | C_17_H_14_O_7_ | 330.07 | Ombuin | | 314.0416;299.0181;271.0237;243.0289 |
| 20 | 26.05 | | [M+FA-H]^-^ | | 957.5071 | 957.5065 | 0.7 | C_47_H_76_O_17_ | 912.51 | 21-Norgypenoside A | | 911.5044;749.4514;603.3953;491.3404 |
| 21 | 26.18 | | [M+FA-H]^-^ | | 975.5536 | 975.5534 | 0.2 | C_48_H_82_O_17_ | 930.56 | β-D-Glucopyranoside,(2α,3β,12β)-2,3,12-trihydroxydammar-24-en-20-ylO-6-deoxy-α-L-mannopyranosyl-(1→2)-O-[6-deoxy-α-Lmannopyranosyl-(1→3)] | | 975.5518;929.5445;783.4871;637.4288;475.3787 |

**Table S3． Sequences of the mouse primers used in real-time PCR**

| Gene | Sense primer | Anti-sense primer |
| --- | --- | --- |
| *β-Actin* | TGTCCACCTTCCAGCAGATGT | AGCTCAGTAACAGTCCGCCTAGA |
| *Pparα* | AGGCTGTAAGGGCTTCTTTCG | GGCATTTGTTCCGGTTCTTC |
| *Pgc1α* | TGTTCCCGATCACCATATTCC | GGTGTCTGTAGTGGCTTGATTC |
| *Tnfα* | ATGGATCTCAAAGACAACCAACTAG | ACGGCAGAGAGGAGGTTGACTT |
| *Mcp-1* | AGGTCCCTGTCATGCTTC | GTGCTTGAGGTGGTTGTG |
| *Il-1β* | TCGTGCTGTCGGACCCATAT | GGTTCTCCTTGTACAAAGCTCATG |
| *Gclm*  *Gclc* | AGGAGCTTCGGGACTGTATCC  GGGGTGACGAGGTGGAGTA | GGGACATGGTGCATTCCAAAA  GTTGGGGTTTGTCCTCTCCC |
| *Gpx*  *Sod2* | CCTTTTAAGCAGTATGCAGGCA  CAGACCTGCCTTACGACTATGG | CAAGCCAAATGGCCCAAGTT  CTCGGTGGCGTTGAGATTGTT |
| *F4/80*  *Acox1* | TGACTCACCTTGTGGTCCTAA  CTTGGATGGTAGTCCGGAGA | CTTCCCAGAATCCAGTCTTTCC  TGGCTTCGAGTGAGGAAGTT |
| *Cpt1α* | TATGTGAGTGACTGGTGGGAGGA | TATGGGTTGGGGTGATGTAGAGC |
| *Chop* | CTCGCTCTCCAGATTCCAGTC | CTTCATGCGTTGCTTCCCA |
| *Casp3* | TGGTGATGAAGGGGTCATTTATG | TTCGGCTTTCCAGTCAGACTC |
